# AKIP1 Expression Modulates Mitochondrial Function in Rat Neonatal Cardiomyocytes

**DOI:** 10.1371/journal.pone.0080815

**Published:** 2013-11-13

**Authors:** Hongjuan Yu, Wardit Tigchelaar, Debby P. Y. Koonen, Hemal H. Patel, Rudolf A. de Boer, Wiek H. van Gilst, B. Daan Westenbrink, Herman H. W. Silljé

**Affiliations:** 1 Department of Cardiology, University Medical Center Groningen, University of Groningen, Groningen, The Netherlands; 2 Department of Hematology, the First Affiliated Hospital of Harbin Medical University, Harbin, China; 3 Molecular Genetics, University Medical Center Groningen, University of Groningen, Groningen, The Netherlands; 4 VA San Diego Healthcare System, San Diego, California, United States of America; 5 Department of Anesthesiology, University of California San Diego, San Diego, California, United States of America; Thomas Jefferson University, United States of America

## Abstract

A kinase interacting protein 1 (AKIP1) is a molecular regulator of protein kinase A and nuclear factor kappa B signalling. Recent evidence suggests AKIP1 is increased in response to cardiac stress, modulates acute ischemic stress response, and is localized to mitochondria in cardiomyocytes. The mitochondrial function of AKIP1 is, however, still elusive. Here, we investigated the mitochondrial function of AKIP1 in a neonatal cardiomyocyte model of phenylephrine (PE)-induced hypertrophy. Using a seahorse flux analyzer we show that PE stimulated the mitochondrial oxygen consumption rate (OCR) in cardiomyocytes. This was partially dependent on PE mediated AKIP1 induction, since silencing of AKIP1 attenuated the increase in OCR. Interestingly, AKIP1 overexpression alone was sufficient to stimulate mitochondrial OCR and in particular ATP-linked OCR. This was also true when pyruvate was used as a substrate, indicating that it was independent of glycolytic flux. The increase in OCR was independent of mitochondrial biogenesis, changes in ETC density or altered mitochondrial membrane potential. In fact, the respiratory flux was elevated per amount of ETC, possibly through enhanced ETC coupling. Furthermore, overexpression of AKIP1 reduced and silencing of AKIP1 increased mitochondrial superoxide production, suggesting that AKIP1 modulates the efficiency of electron flux through the ETC. Together, this suggests that AKIP1 overexpression improves mitochondrial function to enhance respiration without excess superoxide generation, thereby implicating a role for AKIP1 in mitochondrial stress adaptation. Upregulation of AKIP1 during different forms of cardiac stress may therefore be an adaptive mechanism to protect the heart.

## Introduction

A kinase interacting protein 1 (AKIP1) is a small 23 kDa protein originally identified as a breast cancer associated gene (BCA3) [1]. In humans, there are three splice variants, the full-length protein (AKIP1a), one that lacks the third exon (AKIP1b), and one that lacks the third and fifth exon (AKIP1c). In contrast, only the full-length protein is present in rodents [2]. It has no homologies to other proteins, is devoid of particular catalytic domains and is therefore believed to have a role as an adaptor or structural intracellular protein. AKIP1 localizes to the cytoplasm, nucleus, and mitochondria and associations with proteins with different sub-cellular localizations have been reported, including PKA [3], NFκB [4], apoptin [5], RAC1 [6], TAP73 [7] and AIF [8]. These varied sites of cellular localization suggest that AKIP1 may have multiple functions in the cell. In cancer cell lines a role for AKIP1 in nuclear-cytoplasmic shuttling of PKA and NFκB has been proposed [3,4,9,10], but AKIP1 may also be involved in apoptosis [5,7]. AKIP1 has been shown to localize to mitochondria in both cancer cells and cardiomyocytes, but its functional role in mitochondria is still elusive [7,8].

AKIP1 has been mainly studied in cancer cell lines, but is also expressed in many normal, non-tumor, cells in different organs. AKIP1 is abundantly expressed in cardiac tissue predominantly in cardiomyocytes [11]. In a gene array study we identified AKIP1 as a differentially expressed gene that was significantly upregulated in animal models of pathological cardiac hypertrophy and heart failure, including pressure overload and post-myocardial infarction (MI) remodelling [11]; however, exercise mediated “physiological hypertrophy” also increased AKIP1 expression [12]. Cardiac hypertrophy is initially adaptive in cardiomyocytes to compensate for sustained wall stress, but becomes maladaptive during sustained pathological stress. Interestingly, mitochondrial function is improved during physiological hypertrophy, but diminishes upon sustained pathological hypertrophy [13]. It is possible that AKIP1 may be regulating the compensation phase of pathologic hypertrophy and exercise-induced physiologic hypertrophy through regulation of mitochondrial function. Mitochondria isolated from AKIP1 gene transferred hearts showed amongst others, enhanced calcium tolerance, and decreased mitochondrial cytochrome C release upon ischemic stress. Interestingly, AKIP1 overexpression could protect cardiac function in an *ex vivo* mouse ischemia/reperfusion model [8]. Here we test the direct effects of loss or overexpression of AKIP1 on mitochondrial function.

## Materials and Methods

### Ethics statement

Animal use for these studies was in accordance with the NIH Guide for the Care and Use of Laboratory Animals. The study was submitted to, and approved by, the Committee for Animal Experiments of the University of Groningen (Permit Number: DEC6002). All efforts were made to minimize suffering.

### Isolation and culturing of primary cardiomyocytes

Neonatal rats of 1-3 day old were euthanized by decapitation, hearts excised and atria were removed. Primary neonatal rat ventricular cardiomyocytes (NRVCs) were isolated as previously described [14,15]. Cardiomyocytes were grown in DMEM (Sigma D5671, Missouri, USA) supplemented with 5% fetal calf serum (FCS: Sigma F9665, Missouri, USA) and penicillin-streptomycin (100U/ml-100μg/ml; Sigma P0781, Missouri, USA). Adenoviral constructs were generated with the ViraPower^TM^ adenoviral expression system from Invitrogen as described previously [16]. Primers used for cloning are listed in Table S1. For adenoviral infections, cardiomyocytes were infected with an MOI of 25 one day after isolation, in medium with 5% FCS, and starved the next day for 48 hours. Similar culture conditions were employed for the non-infected cells or control virus infected cells that served as controls. For cells stimulated with phenylephrine (PE), 24 hours after starvation PE (50 μM) was added for 24 hours after which assays were performed. 

### Seahorse mitochondrial flux analyses

To measure the rate of oxidative phosphorylation in intact cardiomyocytes, a Seahorse metabolic flux analyzer was used (Seahorse Biosciences, Massachusetts, USA). NRVCs were seeded at a density of 100,000 cells/well in 24 well Seahorse assay plates. Cells were treated as described above. One hour before initiation of measurements, medium was replaced with XF medium supplemented with 10 mM glucose or 1 mM pyruvate and incubated for 1 hour in a 37°C incubator (without CO_2_). Three baseline oxidative consumption rate (OCR) measurements were performed, followed by injection with oligomycin (1 μM) to measure the ATP linked OCR. The uncoupler FCCP (0.5 μM) was used to determine maximal respiration and rotenone (1 μM) and antimycin A (1 μM) were injected to determine the non-mitochondrial respiration. Experimental treatments were performed on 3-4 wells of each plate as technical replicates and each experiment had at least 3 biological replicates. OCR was normalized for the amount of cellular protein in each well.

### Real time PCR

Total RNA was isolated using a kit (Bioke, the Netherlands) and cDNA was synthesized using a reverse transcription kit (Qiagen, the Netherlands) following manufactures instructions. Total DNA was isolated using a kit (Qiagen, the Netherlands) following manufactures instructions. Relative gene expression was determined by quantitative real time PCR (RT-PCR) on a Bio-Rad CFX384 real time system using SYBR Green dye (Bio-Rad, California, USA). Gene expressions were corrected for reference gene values (cyclophilin A or GAPDH), and the calculated values were expressed relative to the control group per experiment. Primer sequences are listed in Table S2. 

### Western blot

Protein was isolated in RIPA buffer (50 mM Tris pH 8.0, 1% nonidet P40, 0.5% deoxycholate, 0.1% SDS, 150 mM NaCl) supplemented with 10 μl/ml phosphatase inhibitor cocktail 3 (Sigma p2850, Missouri, USA), protease inhibitor cocktail (Roche, 11873580001, Switzerland) and 1mM phenylmethylsulfonyl fluoride (PMSF) (Roche,10837091001, Switzerland). Protein concentration was determined with a DC protein assay kit (Bio-Rad, California, USA). Equal amounts of proteins were separated by SDS-PAGE and proteins were transferred to PVDF or nitrocellulose membranes. For detection of specific proteins, the following antibodies were used: a custom-made rabbit polyclonal anti-AKIP1 antibody, anti-total OXPHOS antibodies cocktail (Abcam, Cambridge, United Kingdom), and monoclonal anti-GAPDH antibody (Fitzgerald, Massachusetts, USA) served as a loading control. After incubation with secondary antibodies, signals were visualized with ECL and analyzed by densitometry (Syngene, Cambridge, United Kingdom).

### Citrate synthase assay

Whole cell citrate synthase activity was measured using an enzyme assay kit (Sigma, Missouri, USA) according to the manufactures instructions. Cell lysates were prepared using the CelLytic M Cell Lysis Reagent and protein concentrations were measured with a DC protein assay kit (Bio-Rad, California, USA). 8 μg of protein was mixed with Acetyl CoenzymeA (Acetyl CoA), 5,5’-Dithiobis-(2-nitrobenzoic acid) (DTNB), and assay buffer in a 96 well plate. The reaction was initiated by adding Oxaloacetate (OAA) into the mixture and total activity was measured. Absorbance was measured at 412 nm using a multiwall spectrophotometer following a kinetic program. Triplicate measurements were performed on each sample and 5 independent samples were measured for each group.

### Determination of mitochondrial membrane potential and ROS production

Mitochondrial membrane potential (ΔΨm) was assessed by tetramethylrhodamine ethyl ester (TMRE; Life Technologies Europe BV, The Netherlands) following the manufacturers instructions. In brief, cells were seeded at a density of 40,000 cells/well in black 96 well plates and cultured as described above. TMRE was added at a final concentration of 100 nM with 20 min incubation at 37 °С. Cells were subsequently washed with 0.2% BSA in PBS, and fluorescence was measured at 575 nm with a SynergyH4 Hybrid Reader (BioTek, Vermont, USA). As a control, FCCP (1 μM) was added to some wells. ROS production was determined in a similar way using MitoSOX red mitochondrial superoxide indicator (Life Technologies, California, USA). Cells were plated in 96 well plates and treated as above. On the day of measurement, the cells were incubated with MitoSOX (5 μM) in KRPH buffer (20mM HEPES, 5 mM KH_2_PO_4_, 1 mM MgSO_4_, 1 mM CaCL_2_, 136 mM NaCl, 4.7 mM KCl, pH 7.4) for 10 min at 37 °С, and thereafter washed three times with the same buffer. Fluorescence was measured using excitation/emission maxima of 510/580 nm with a SynergyH4 Hybrid Reader (BioTek, Vermont, USA). For both assays, each experiment had four technical and three biological replicates.

### Statistical analysis

All values are presented as mean ± standard error of the mean (SEM). Independent-samples t-test was performed to compare the difference between both groups. One-way ANOVA with post-hoc test was used to compare the difference between 4 groups with homogeneity of variances. Kruskal-Wallis test was used to compare the difference between 4 groups with nonparametric variances. P<0.05 was considered to be significant. SPSS software (PASW Statistics 18) was used in the statistics analysis. 

## Results

### AKIP1 silencing attenuates PE induced OCR

**Figure 1:**
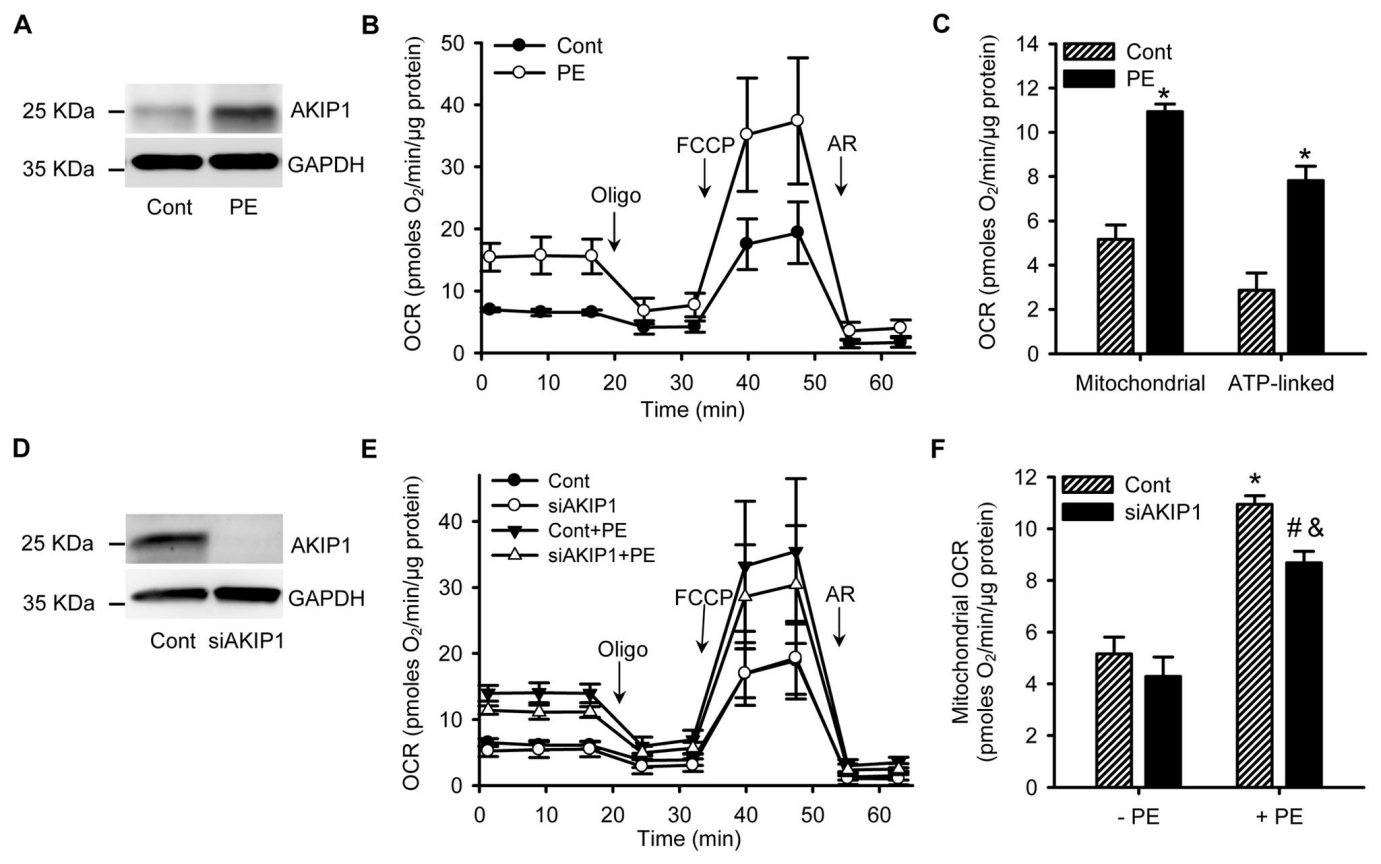
AKIP1 silencing attenuates PE-induced OCR. A. A representative Western blot is shown with control and PE treated NRVCs samples. Blots were probed with anti-AKIP1 antibody and anti-GAPDH as a loading control. B. PE induced OCR in NRVCs. Metabolic flux in cardiomyocytes was determined with a Seahorse flux analyzer in the presence of 10 mM glucose. Addition of the ATP synthase inhibitor oligomycin (oligo), the uncoupler FCCP and the respiratory chain inhibitors antimycin A and rotenone (AR) are indicated. C. Mitochondrial OCR (difference between basal and AR treated levels) and ATP-linked OCR (difference between basal and oligomyin treated levels) are shown. OCR was corrected for total protein levels in each well (pmoles O_2_/min/μg) (*P<0.05 as compared to cont, n=4). D. A representative Western blot is shown of cells treated for 72 hours with control and siAKIP1 adenovirus. Blots were probed with anti-AKIP1 antibody and anti-GAPDH as a loading control. E. Silencing of AKIP1 can limit PE induced OCR. OCR was measured as above. F. Quantification of mitochondrial OCR showed significant lower OCR in siAKIP1+PE group compared to Cont+PE group (*P<0.05 as compared to cont group, #P<0.05 compared to siAKIP1 group, &P<0.05 compared to cont+PE group, n=4). All values are presented as mean ± SEM.

We have recently shown that AKIP1 gene expression can be induced by PE, a pharmacologic inducer of hypertrophy [11]. We confirm this finding in the current data and, moreover, show that AKIP1 protein expression is induced by PE in NRVCs (Figure 1A). PE has been shown to increase respiration in NRVCs [17]. We wondered whether AKIP1 plays a role in modulating PE-induced mitochondrial respiration. Addition of PE to NRVCs resulted in a marked increase in OCR (doubling in OCR, see Figure 1B). Addition of oligomycin (oligo), an ATP-synthase inhibitor, reduced OCR both in control and PE treated cells. This difference in OCR between basal and oligomycin treated conditions, is the ATP-linked OCR, which was higher in the PE treated cells (Figure 1C). Subsequent addition of FCCP, an uncoupling agent, provided the maximal, uncoupled, OCR. Finally, antimycin-A and rotenone (AR) were added to the cells to block mitochondrial respiration (complex III and complex I inhibitors, respectively). The basal OCR was subsequently corrected for this non-mitochondrial OCR to obtain the mitochondrial specific respiration, which was significantly elevated in PE treated cells (Figure 1C). To investigate whether this increase in mitochondrial respiration required AKIP1, we used an adenoviral small interfering RNA system to silence AKIP1. Silencing of AKIP1 resulted in a 90% reduction of AKIP1 protein levels (Figure 1D). Interestingly, AKIP1 silencing reduced, albeit not significantly, mitochondrial OCR in NRVCs (Figure 1E, F). Upon PE addition, this reduction was much more pronounced and mitochondrial OCR was significantly attenuated (Figure 1E, F). AKIP1 silencing could also inhibit PE induced ATP-linked OCR, albeit just not significant (Figure S1). Thus, AKIP1 silencing attenuated PE induced mitochondrial OCR, indicating a role for AKIP1 in mitochondrial respiration.

### AKIP1 induces mitochondrial OCR

**Figure 2:**
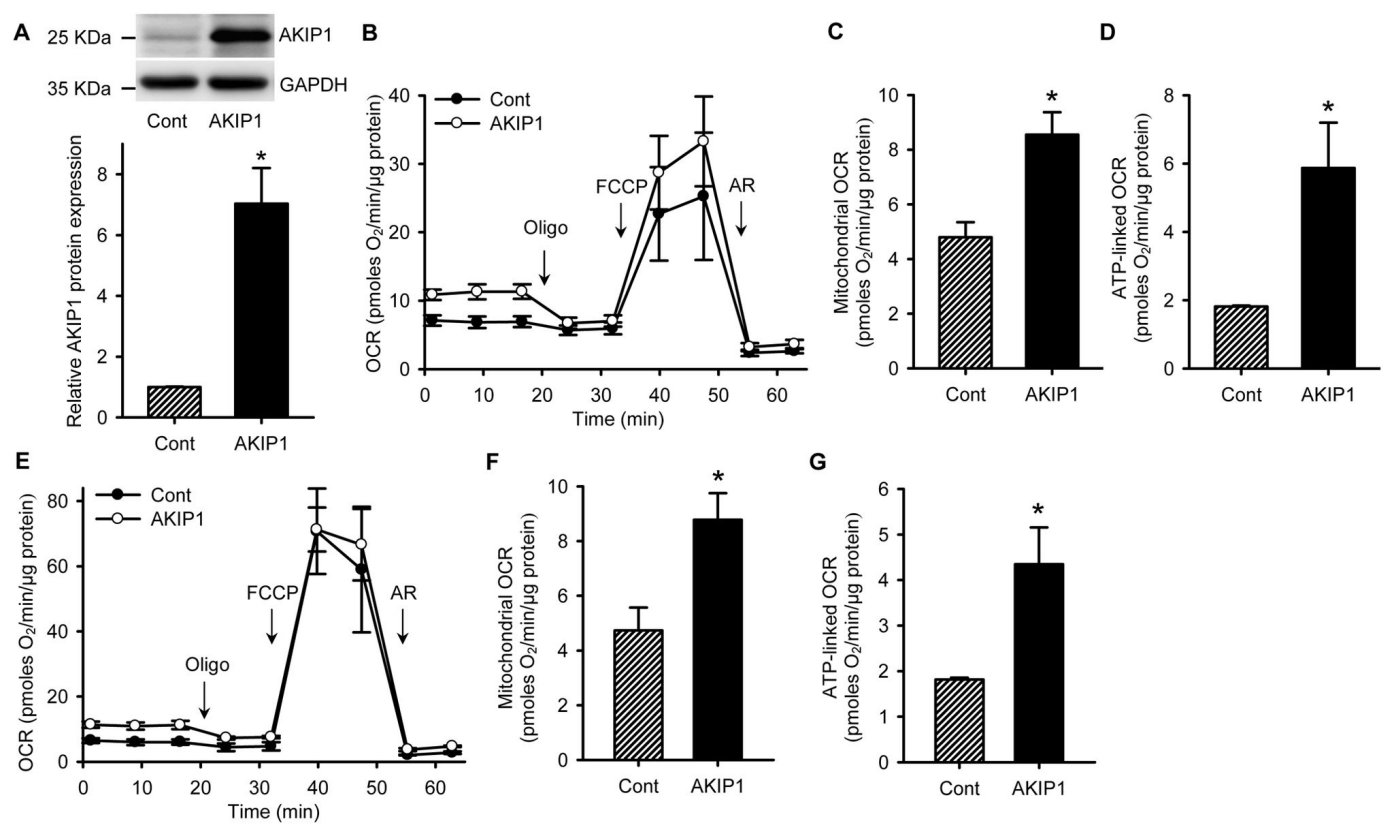
AKIP1 overexpression induces mitochondrial OCR. A. Using an adenoviral construct, rat AKIP1 was overexpressed in NRVCs. Western blots of control and AKIP1 overexpressing cells were probed with anti-AKIP1 antibody and anti-GAPDH as a loading control. Quantification of 3 blots is shown in the bar diagram (*P<0.05 as compared to cont group). B. AKIP1 induced OCR in NRVCs. Metabolic flux in cardiomyocytes was determined in the presence of 10 mM glucose. Addition of different inhibitors is indicated. C. Mitochondrial OCR of control and AKIP1 overexpressing cells is shown. D. ATP-linked OCR of control and AKIP1 overexpressing cells is shown. In all cases OCR was corrected for total protein levels in each well (pmoles O_2_/min/μg) (*P<0.05 as compared to cont group, n=8). E-G. AKIP1 induced OCR in NRVCs in the presence of 1 mM pyruvate. The experiment was performed as above, but using pyruvate as substrate instead of glucose (*P<0.05 as compared to cont group, n=4). All values are presented as mean ± SEM.

To investigate whether AKIP1 itself could induce mitochondrial OCR, we overexpressed AKIP1 in NRVCs (Figure 2A). AKIP1 increased basal OCR by almost 80 % (Figure 2B). Blocking the mitochondrial respiratory chain with AR almost fully blocked this OCR, suggesting that this was specific for mitochondrial respiration. The calculated mitochondrial specific OCR was 4.8 pmoles O_2_/min/μg protein in control and 8.5 pmoles O_2_/min/μg protein in AKIP1 overexpressing cells (Figure 2C). The oligomycin effects on OCR suggested the increase was linked to ATP synthesis (Figure 2D). Also, the maximal respiratory rate was increased, as determined by FCCP uncoupling; however, this was not significant. The reserve capacity, the difference between maximal and basal respiration, remained similar (Figure 2B). To further investigate the role of AKIP1 in mitochondrial oxidation and to test whether this was independent of the glycolytic flux we performed a similar experiment with pyruvate as a substrate. Pyruvate can be converted into acetyl-CoA in mitochondria and directly be used as a substrate in the TCA cycle. Interestingly, with pyruvate similar results were obtained as with glucose (Figure 2E-G), indicating that these changes in mitochondrial respiration were independent of glycolytic flux. This was further confirmed by the absence of changes in extracellular acidification rate in these cells. 

### AKIP1 does not induce mitochondrial biogenesis

**Figure 3:**
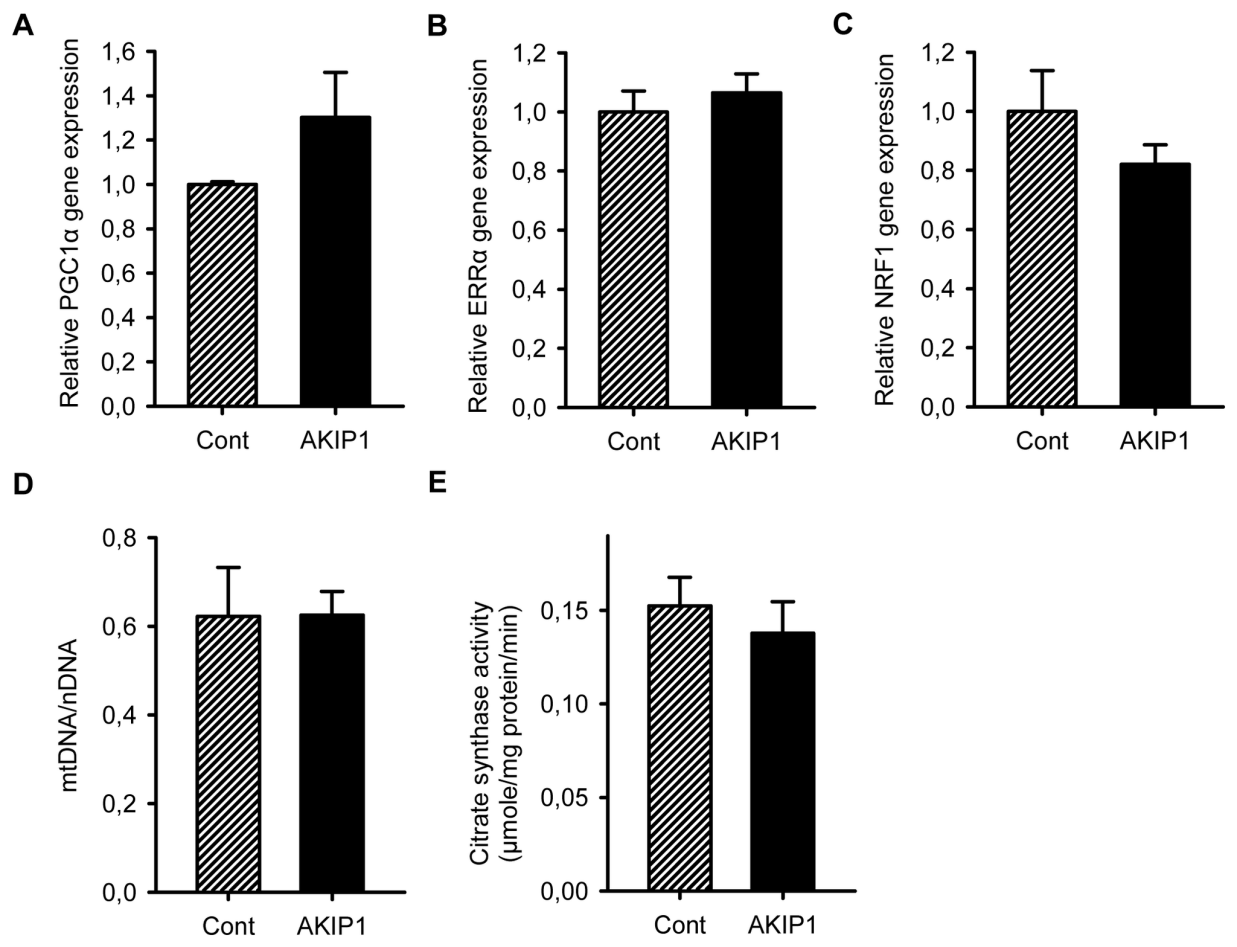
AKIP1 does not induce mitochondrial biogenesis. A-C. Expression of mitochondrial biogenesis controlling genes PGC1α , ERRα and NRF1 was determined by RT-PCR in control and AKIP1 overexpressing NRVCs. Relative expression levels were normalized by expression levels of the household gene, cyclophilin A. No significant difference was observed between groups (n=3-6). D. Nuclear DNA (TRPM-2) to mitochondrial DNA (CYTB) ratio was determined by RT-PCR on DNA isolated from control and AKIP1 overexpressing cells. No statistical significant difference was observed between groups (n=3). E. Citrate synthase activity was measured on whole cell lysates from control and AKIP1 overexpressing cardiomyocytes as described in method section. No statistical significant difference was observed between groups (n=5). All values are presented as mean ± SEM.

AKIP has been shown to modulate SIRT1 function, and the latter has been shown to control the expression of PGC1α, a master transcriptional regulator for mitochondrial biogenesis. Therefore, AKIP1 induced OCR could potentially be a result of an increase in mitochondrial biogenesis. In AKIP1 overexpressing cells PGC1α was not significantly increased (Figure 3A). Also expression of ERRα and NRF1, downstream targets of PGC1α involved in the regulation for mitochondrial biogenesis were not altered (Figure 3B, C). To corroborate this finding, we also determined the ratio between mitochondrial DNA (mtDNA) versus nuclear DNA (nDNA), a generally accepted measurement of mitochondrial density. PCR analysis was performed on the nuclear gene TRPM-2 and the mitochondrial gene CYTB. The ratio between mtDNA and nDNA was similar in control and AKIP1 overexpressing cells indicating that mitochondrial biogenesis was not increased (Figure 3D). To further confirm this by an independent method we assessed citrate synthase activity which was not affected by AKIP1 overexpression (Figure 3E). Multiple data support the notion that AKIP1 does not stimulate mitochondrial biogenesis. 

### No increase in OXPHOS levels in AKIP1 overexpressing cells

**Figure 4:**
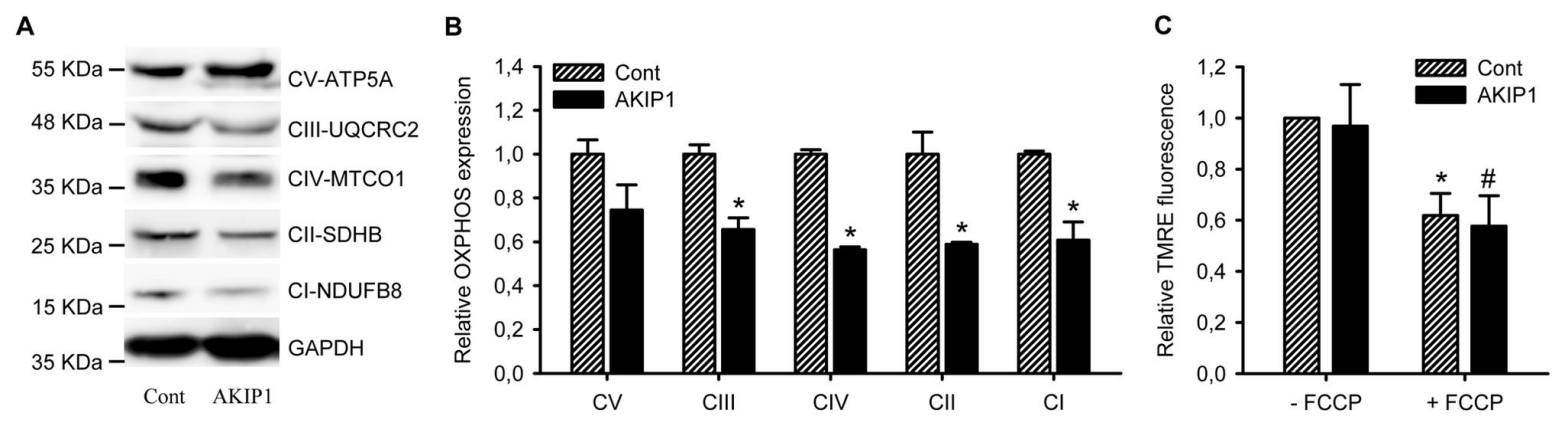
OXPHOS levels are reduced in AKIP1 overexpressing cells. A. Western blot analysis was performed on proteins isolated from control and AKIP1 overexpressing NRVCs, anti-total OXPHOS antibody cocktail was used to determine ETC protein levels. GAPDH was used as loading control. Representative blot is shown. B. Quantification of 4 independent experiments is shown in the bar diagram (*P<0.05 as compared to cont, n=4). C. Mitochondrial potential was measured using TMRE in control and AKIP1 overexpressing cells. As a control the ionophore FCCP was added to reduce membrane potential (*P<0.05 as compared to cont, #P<0.05 as compared to AKIP1 group, n=4). All values are presented as mean ± SEM.

The altered respiratory rates could also result from changes in expression of electron transport chain (ETC) complexes. Western blotting with an OXPHOS antibody cocktail, recognizing multiple subunits of the ETC, did not show an increase in the protein levels of ETC-complexes (Figure 4A, B). On the contrary, a significant decrease could be observed in subunits of complex I, II, III and IV in AKIP1 overexpressing cells. Also complex V appeared to be somewhat reduced, albeit not significant. This suggests that AKIP1 overexpression enhances the efficiency of mitochondrial ETC. We also analyzed changes in mitochondrial membrane potential using the fluorophore TMRE. As shown in Figure 4C, no significant difference between control and AKIP1 infected cells was observed in TMRE fluorescence. Treatment with FCCP, a depolarizing agent did strongly reduce TMRE signal, confirming membrane potential specificity (Figure 4C). Thus despite lower OXPHOS levels, AKIP1 overexpressing did not significantly affect the mitochondrial membrane potential, suggesting a more efficient mitochondrial respiration. 

### AKIP1 reduces superoxide formation

**Figure 5:**
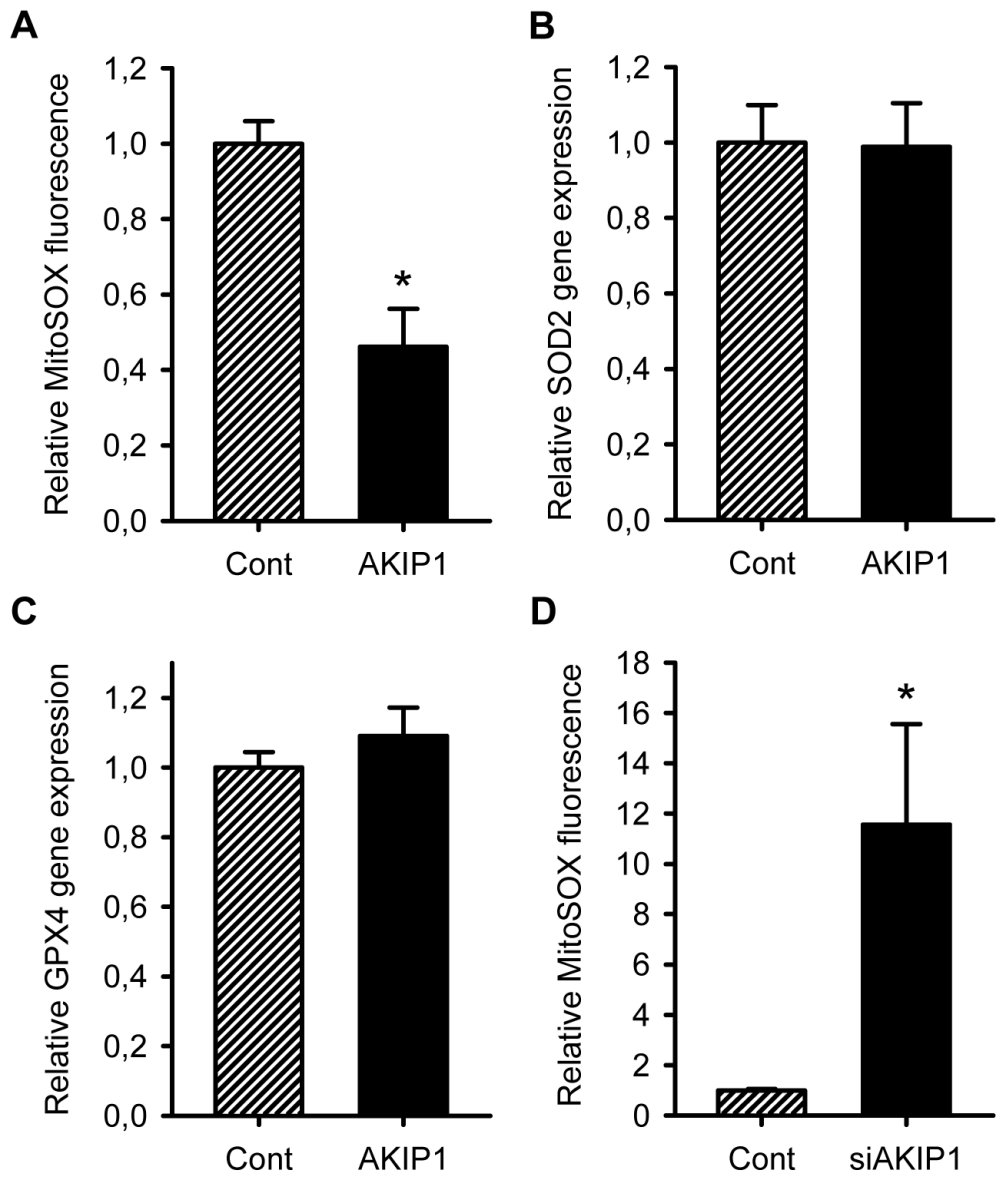
AKIP1 reduces superoxide formation. A. Mitochondrial ROS was determined using MitoSOX fluorophore in control and AKIP1 overexpressing cells. Significant less ROS signal was observed in AKIP1 group (*P<0.05 compared to cont, n=6). B and C. Anti-oxidant genes SOD2 and PGX4 were determined by RT-PCR, relative gene expression was normalized by expression levels of the household gene, cyclophillin A. No significant differences were observed between groups (n=6). D. Mitochondrial ROS was determined as above in control and AKIP1 silenced cells. Significantly increased ROS signal was observed in the AKIP1 silenced cells (*P<0.05 compared to cont, n=4). All values are presented as mean ± SEM.

Since OCR was elevated in AKIP1 overexpressing cells, we analyzed whether the potential of mitochondrial to generate reactive oxygen species would be altered. We assessed superoxide generation using MitoSOX, a fluorophore which is activated by superoxide oxidation. Interestingly, MitoSOX signal in AKIP1 overexpressing cells was about half as compared to control cells (Figure 5A). To exclude that this could be due to increased levels of oxygen radical scavenging systems, we also analysed the expression of manganese-superoxide dismutase (SOD2) and glutathione peroxidise (GPX4). Neither of these genes were upregulated (Figure 5B, C), indicating that AKIP1 does not induce the anti-oxidant response. In contrast, AKIP1 silencing resulted in increased ROS production (Figure 5D). Together, this suggests that AKIP1 overexpression improves mitochondrial function to enhance respiration without excess generation of ROS implicating a role for AKIP1 in mitochondrial stress adaptation. 

## Discussion

AKIP1 protein is expressed in many organs, and in cardiac tissue a role in mitochondrial function has been suggested [8]. Although its role at mitochondria is still elusive, AKIP1 localizes to and fractionates with mitochondria [7,8]. Here we investigated the effect of AKIP1 in mitochondrial respiration in neonatal rat cardiomyocytes. Treatment of cells with PE induced AKIP1 expression and increased OCR. Interestingly, AKIP1 overexpression on its own was sufficient to induce mitochondrial OCR, whereas AKIP1 silencing could attenuate PE induced OCR. We further determined that these effects were independent of mitochondrial biogenesis and changes in ETC component density. The increase in respiratory function was not associated with increased ROS formation, suggesting that AKIP1 overexpression may be a means to enhance the efficiency of mitochondrial function in setting of cardiac stress. Having effects independent of structural changes to mitochondria that could take time to manifest (i.e., biogenesis and protein synthesis to increase ETC components) suggest that localization and stress induced expression and/or translocation of AKIP1 may be important regulatory functions of AKIP1 in the cell. 

In concordance with a previous study [17], we showed that PE induced mitochondrial respiration in cardiomyocytes. PE also induced AKIP1 expression and silencing of AKIP1 could attenuate PE induced alteration in mitochondrial function. Interestingly, AKIP1 overexpression was itself sufficient to increase OCR. Since oxygen consumption was also increased with pyruvate as a substrate, this was independent of potential glycolytic effects. Together these data indicate that AKIP1 has a contributory role in PE induced mitochondrial respiration. Since AKIP1 localizes to the nucleus and has been shown to modulate transcription via NFκB and SIRT1 in cancer cell lines [10,18], the mitochondrial changes may be a result of altered transcription of nuclear encoded mitochondrial genes. We investigated expression of numerous genes encoding mitochondrial proteins, but did not observe any obvious differences. SIRT1 modulation has also been shown to affect PGC1α transcription, a well-known activator of mitochondrial biosynthesis [19]. PGC1α and other downstream mitochondrial biosynthesis genes, like ERRα and NRF1, were not significantly altered. Moreover, mitochondrial numbers were not increased in these cells. Thus, although we cannot rigorously exclude the possibility that AKIP1 modulates mitochondrial function via transcriptional regulation, we currently find this explanation unlikely. 

In AKIP1 overexpressing cells lower levels of OXPHOS components were observed. Nevertheless, mitochondrial membrane potential was similar as compared to control cells and oxygen consumption rate was higher. The flux per ETC complex must therefore be considerably higher in AKIP1 overexpressing cells accounting for the higher efficiency. ROS production on the other hand was attenuated suggesting that the enhanced respiration was coupled and resulted in energy production rather than mitochondrial dysfunction. Diminished ROS production has recently been observed in mitochondria from AKIP expressing adult cardiomyocytes, further stressing that this is a mitochondrial specific effect [8]. Within mitochondria at least ten different enzymes contribute to ROS formation, but ETC complexes I and III are believed to be the major sites of superoxide production [20-22]. Moreover, as we observed changes in the levels of components of these complexes, it is likely that AKIP1 exerts its effects by modulating ETC in some way. It is possible that AKIP1 interacts with mitochondrial localized proteins, possibly components of the ETC, to enhance function and decrease ROS generation. Nevertheless, other factors like mitochondrial ultrastructural or morphological changes in AKIP1 overexpressing cells, might also contribute to changes in mitochondrial function. Cardiac hypertrophy and failure are also associated with changes in multiple components of the ETC. However, even within the same model and study, divergent responses in different ETC components have been observed, underscoring the complex regulatory networks that controls cardiac energy production [23-25]. How AKIP1 modulates the levels and function of these complexes will therefore require further investigations. 

**Figure 6:**
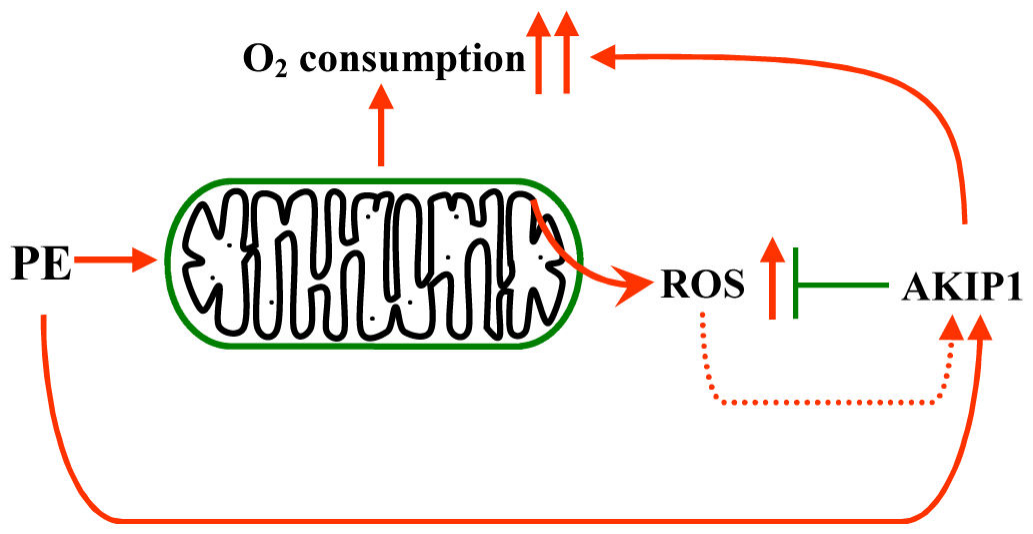
Schematic model of the role of AKIP1 in modulating OCR and ROS formation. PE induced OCR and induced ROS formation in mitochondria. PE also induced AKIP1 expression and AKIP1 was able to attenuate ROS formation, but stimulate OCR. AKIP1 silencing experiments confirmed this role of AKIP1 in controlling OCR and ROS production. Together this indicates that AKIP1 improves mitochondrial function.

Conditions that trigger hypertrophy and heart failure formation, as well as ischemia/reperfusion, trigger AKIP1 expression in the heart [8,11]. Increased AKIP1 levels result in more efficient mitochondrial respiration and reduced ROS formation. However, despite the presence of higher AKIP1 levels under these conditions, decreased mitochondrial function and increased ROS formation has been reported with multiple forms of cardiac stress [26-28]. Also, in PE treated cardiomyocytes ROS production is increased [29], despite increased AKIP1 expression. This apparent contradiction might be explained by the fact that all these conditions result in profound metabolic and mitochondrial changes. The increase in AKIP1 expression under these conditions might therefore be an adaptive response to limit ROS production (Figure 6). This is comparable with genes like SOD and catalase, which encodes enzymes that scavenge ROS and are expressed only when ROS levels are high [30]. It will therefore also be interesting, whether absence of AKIP1 makes cells more vulnerable to cardiac stress conditions. Alternatively, since increased AKIP1 expression has also been reported during physiological hypertrophy (exercise), AKIP1 expression may be induced to enhance mitochondrial performance during cardiac stress events. Many factors that are shown to be compensatory in heart failure (e.g., natriuretic peptides, caveolins, etc) show initial patterns of upregulation that ultimately lead to heart failure, however, these same factors have a potential protective role in heart failure when they are overexpressed [31-33]. Such may be the case for AKIP1 expression. During sustained stress, other metabolic and mitochondrial alterations might subsequently result in deterioration of mitochondrial function and increased ROS production. Obviously this still begs the question, why AKIP1 expression is not continuously high, since improved mitochondrial respiration would be beneficial under all conditions. There could be multiple reasons; first depending on its localization AKIP1 has also different functions besides regulating mitochondrial respiration. Moreover, ROS is a double-edged sword. High levels are detrimental causing DNA and protein damage, whereas low levels are beneficial and are required for cell signalling [28,34]. Therefore sustained high expression of AKIP1 under normal conditions might interfere with other cellular processes.

All together, we have shed light on the role of AKIP1 in cardiomyocyte energy generation. In particular, we have shown that AKIP1 can improve mitochondrial function in cardiomyocytes resulting in increased respiration without enhanced ROS production. Activating AKIP1 could therefore have cell protective effects, whereas interfering with its function may be detrimental.

## Supporting Information

Figure S1 **AKIP1 silencing and ATP-linked OCR.** OCR was measured as described in the materials and methods and calculation of ATP-linked OCR was similar as described in Figure 1C. (*P<0.05 as compared to cont group, #P<0.05 compared to siAKIP1 group, n=4). Values are presented as mean ± SEM.
(TIF)

Table S1 **Primers used for cloning.**
(PDF)

Table S2 **Primers used for Real-Time PCR.**
(PDF)
