# Supplementary figures and images for: AKIP1 Expression Modulates Mitochondrial Function in Rat Neonatal Cardiomyocytes
Source: PLoS One. 2013 Nov 13;8(11):e80815. doi: 10.1371/journal.pone.0080815 (PMC3827472; doi:10.1371/journal.pone.0080815)

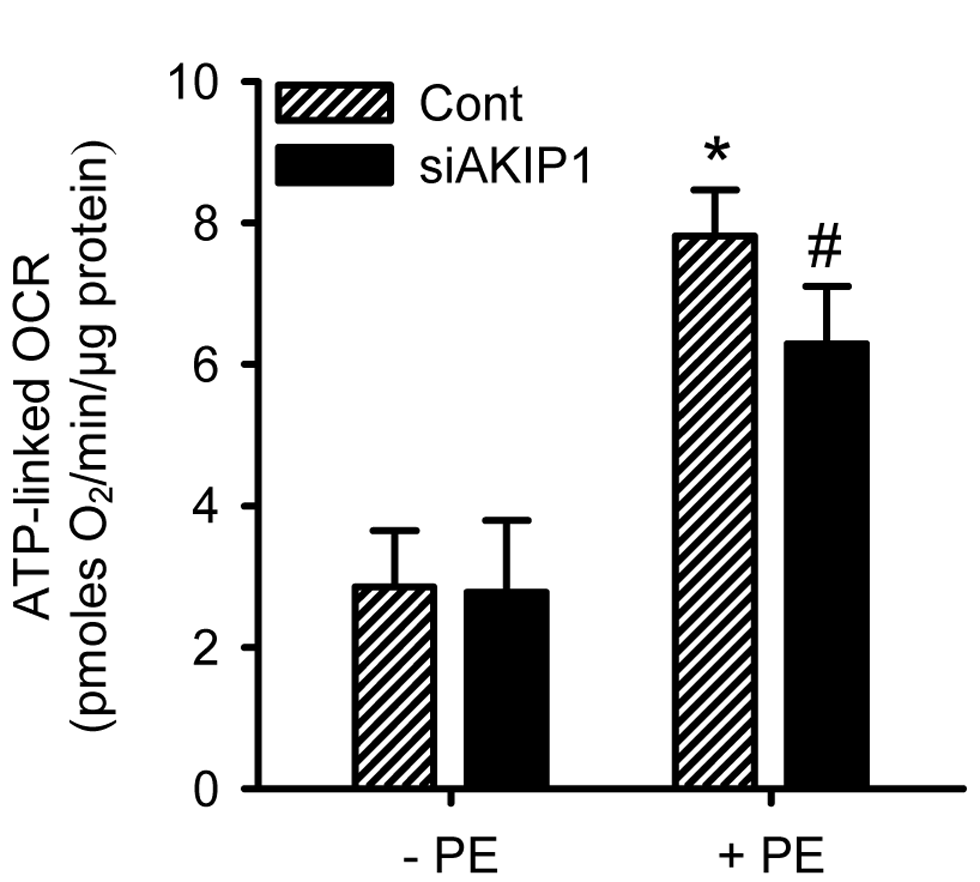

Supplement: Figure S1 — AKIP1 silencing and ATP-linked OCR. OCR was measured as described in the materials and methods and calculation of ATP-linked OCR was similar as described in Figure 1C. (*P<0.05 as compared to cont group, #P<0.05 compared to siAKIP1 group, n=4). Values are presented as mean ± SEM. (TIF) [file pone.0080815.s001.tif]
