# Supplementary material for: AKIP1 Expression Modulates Mitochondrial Function in Rat Neonatal Cardiomyocytes
Source: PLoS One. 2013 Nov 13;8(11):e80815. doi: 10.1371/journal.pone.0080815 (PMC3827472; doi:10.1371/journal.pone.0080815)
Supplement: Table S1 — Primers used for cloning. (PDF) [file pone.0080815.s002.pdf]

Table S1. Primers used for cloning.

**Primers**	**5’-3’**
AKIP1-foward	GAAGGATCCGTCGACATGGAATACTGCCTGGCGGC
AKIP1-reverse	GAACTCGAGTCATACGGGGAACACCAAGTCCAC
siAKIP1-forward	GATCCCGTGGTTGCAGTTGACTCGTTCAAGAGAGACCGAGTCAACTGCAACCACTTTTTGGAAA
siAKIP1-reverse	AGCTTTTCCAAAAAGTGGTTGCAGTTGACTCGGTCTCTTGAACCGAGTCAACTGCAACCAACGG
