# Supplementary material for: AKIP1 Expression Modulates Mitochondrial Function in Rat Neonatal Cardiomyocytes
Source: PLoS One. 2013 Nov 13;8(11):e80815. doi: 10.1371/journal.pone.0080815 (PMC3827472; doi:10.1371/journal.pone.0080815)
Supplement: Table S2 — Primers used for Real-Time PCR. (PDF) [file pone.0080815.s003.pdf]

Table S2. Primers used for Real-Time PCR.

Genes	5'-3' forward	5'-3' reverse
Cyclophilin A	CAGATCGAGGGATCGATTCAG	TCACCACTTGACACCCTCATTC
AKIP1	TGGTCCAGGAAGCATCTATC	CAACCACATGCGTCTTCTTG
PGC1α	ACCGTAAATCTGCGGGATGATG	CATTCTCAAGAGCAGCGAAAGC
ERRa	GTGGCCGACAGAAGTACAAG	GGTTCAACCACCAGCAGATG
NRF1	TTGGAGCACTTACTGGAGTC	CTTCCGCCATAATGAATCCC
TRPM-2	GTACAACGAGCTGCTTCATTCC	GCACCTCTAAGAGGCATCCATC
CYTB	CCTCCCATTCATTATCGCCGCCCTTGC	GTCTGGGTCTCCTAGTAGGTCTGGGAA
SOD2	TGGCTTGGCTTCAATAAGGA	AAGGTAGTAAGCGTGCTCCCACAC
GPX4	GCTGTGCGCGCTCCAT	CCATGTGCCCGTCGATGT

PGC1α: Peroxisome proliferator-activated receptor gamma coactivator 1-alpha; ERR α: Estrogen related receptor alpha; NRF1: Nuclear respiratory factor 1; TRPM-2: Testosterone-repressed prostate message-2; CYTB: Cytochrome b, mitochondrial; SOD2: Superoxide dismutase 2; GPX4: Glutathione peroxidase 4.
